# Hospital Reform

**Published:** 1897-10-30

**Authors:** 


### The Hospital

A Journal of

The Medical Sciences and Hospital Administration.

Vol. XXIII.—No. 579.] [Oct. 30, 1897.

### Hospital Reform.

That very well-meaning body of persons, the Hospital Reform Association, and their indefatigable secretary, Mr. Garrett Horder, appear to be quite seriously bent upon convincing the public that the hospital arrangements of the present day leave much to be desired, and that they are in many respects sources of demoralisation to patients, and of loss to the medical practitioners of their respective neighbourhoods. Perhaps, with exceptions in favour of certain institutions, both of these propositions were very generally admitted by the speakers at the meeting convened by the Association last week; but neither on that occasion, nor in the publications on the subject issued by the Hospital Committee of the Charity Organisation Society, do we find any very definite or workable proposals for amendment. The suggestion of some sort of general Board, or other central body, which shall be put in possession of funds from some as yet undiscovered source, and which, in the administration of these funds, shall encourage virtue and discourage vice, in so far as such opposing conditions can be predicated of hospital management, does not seem to have been at all generally approved; and it is said that the authorities of the great hospitals, against which in all probability the smallest amount of mismanagement could be established, would be absolutely opposed to any course which might bring them under any kind of collective jurisdiction. These authorities probably hold that the charges brought against hospital administration are at best greatly exaggerated, and that the alleged abuses are either non-existent, or existent only in the imaginations of critics who are unaccustomed to conduct their mental operations under the control of facts. As long as feelings of this kind exist, as long as reformers pronounce hospitals to be altogether bad, while those who manage them pronounce them to be altogether good, it will be impossible to arrive at any agreement either as to the changes which are required, or as to the manner in which they could be brought about.

It would clearly be a great step towards the settlement of a discussion which certainly does hospitals no good, and which may easily do them incalculable harm, if both sides could agree upon some method by which the truth could be ascertained, and by which, while any flagrant abuses were rooted out at once, any which were less flagrant, but which still became apparent, on full examination, to impartial persons of sound judgment, might be gradually dealt with in a proper manner. We think it would be quite possible to establish a tribunal by which both these ends would be accomplished. In the first place, it would be generally conceded, even by the most advanced reformers, that existing hospitals may be divided into two great classes; namely, those which are required for the treatment of the sick poor and for the conduct of medical education—those, in short, which are necessary to the well-being of society, and of which nothing worse could be said than that they might be improved as regards certain details of management; and those which might with advantage be abolished, either because they are altogether superfluous, or because the vices of their administration appear to be incurable. It will also be conceded that the liability to assessment for local rates constitutes a very serious burden upon hospitals, and one which it would be the duty of the management to escape, if any means of doing so were open to them.

In view of these facts, we submit as a basis for discussion, without in any way committing ourselves to them, the following suggestions made by an influential hospital surgeon. They should be read in conjunction with the article on "The Outpatient Problem," on p. 85:—"All hospital reformers might unite in seeking an enactment by which hospitals should be exempted from rates under the fulfilment of certain conditions. The decision in every case should rest with some impartial authority appointed for the purpose, the proceedings of which should be so directed as to bring about a gradual tightening of the reins of management in the direction of the control of abuses. For the educational hospitals, those attached to the great medical schools, perhaps the General Medical Council, would form the best tribunal; but it would be alike too cumbrous and too costly for smaller and for provincial institutions, which might be referred to committees of the local magistracy, or to County Councils, all such bodies being guided by the decisions of the Medical Council in the cases falling directly under its jurisdiction. If any possibility of escaping rates by obtaining the approval of a competent tribunal were once established, the managers of every hospital, real or so-called, would be bound to endeavour to secure the privilege for the institution under their control; and thus, in a comparatively short time, hospitals would come to be divided into three classes, those which obtained the privilege, those which sought but failed to obtain it, and those which were so conscious of shortcomings as not even to seek it. The first class would be able to appeal confidently to the public for support; while the two latter classes would stand condemned as being either superfluous or ill-managed. A good hospital saves much money to the rates of the place in which it is situate, and thus the adjoining ratepayers, each of whom would pay some infinitesimally small sum on account of the exemption, would after all be gainers in a pecuniary sense. It would be obviously impossible, in the present state of public opinion, or rather of public ignorance, to constitute any central authority to which the authorities of hospitals would find it advantageous to submit themselves voluntarily; but it would be quite within the province of the legislature to confer a benefit, and at the same time to lay down the conditions necessary to its enjoyment."

